# Ethanolic Extracts of *Datura innoxia* Have Promising Acaricidal Activity against *Rhipicephalus microplus* as It Blocks the Glutathione S-Transferase Activity of the Target Tick

**DOI:** 10.3390/genes14010118

**Published:** 2022-12-31

**Authors:** Saman Saman, Chien-Chin Chen, Nosheen Malak, Afshan Khan, Nasreen Nasreen, Adil Khan, Sadaf Niaz, Gauhar Rehman, Roger I. Rodriguez-Vivas, Raquel Cossío-Bayúgar

**Affiliations:** 1 Department of Zoology, Abdul Wali Khan University Mardan, Mardan 23200, Pakistan; 2 Department of Biotechnology and Bioindustry Sciences, College of Bioscience and Biotechnology, National Cheng Kung University, Tainan 701, Taiwan; 3 Department of Pathology, Ditmanson Medical Foundation Chia-Yi Christian Hospital, Chiayi 600, Taiwan; 4 Department of Cosmetic Science, Chia Nan University of Pharmacy and Science, Tainan 717, Taiwan; 5 Department of Zoology, Bacha Khan University Charsadda, Charsadda 24631, Pakistan; 6 Departamento de Salud Animal y Medicina Preventiva, Facultad de Medicina Veterinaria y Zootecnia, Universidad Autónoma de Yucatán, Merida 97000, Yucatán, Mexico; 7 Departamento de Artropodología, Centro Nacional de Investigaciones Disciplinarias en Salud Animal e Inocuidad, Instituto Nacional de Investigaciones Forestales Agrícolas y Pecuarias (INIFAP), Boulevard Cuauhnahuac No. 8534, Jiutepec 62574, Morelos, Mexico

**Keywords:** *Datura innoxia*, acaricide, glutathione S-transferases, phytochemicals, docking studies, tick-borne disease

## Abstract

*Rhipicephalus microplus* is a major bovine ectoparasite that negatively impacts the cattle industry. The acaricidal activity of *Datura innoxia* ethanolic plant extract against *R. microplus*, compared with trichlorfon, was examined using the adult immersion test (AIT), and larval packet test (LPT). In vitro acaricidal activity of the selected plant extract against *R. microplus* engorged females was evaluated at different concentrations (2.5, 5, 10, 20, and 40 mg/mL), and was the same for AIT and LPT. It was further supported by in silico molecular docking of *D. innoxia*’s 21 phytochemicals against the *R. microplus* Glutathione S-transferases (RmGST) protein’s three-dimensional (3D) structure predicted by the trRosetta server. The modeled 3D structure was then evaluated and confirmed with PROCHECK, ERRAT, and Verify3D online servers. To predict the binding mechanisms of these compounds, molecular docking was performed using Auto dock Vina software, and molecular dynamic (MD) simulations were used to investigate the protein atom’s dynamic motion. *D. innoxia* has a relatively higher inhibitory effect on oviposition (from 9.81% to 45.37%) and total larval mortality (42.33% at 24 h and 93.67% at 48 h) at 40 mg/mL. Moreover, the docking results showed that the chemicals norapoatropine and 7-Hydroxyhyoscyamine have strong interactions with active site residues of the target protein, with a docking score of −7.3 and −7.0 Kcal/mol, respectively. The current work also provided a computational basis for the inhibitors of Glutathione S-transferases that were studied in this research work, and this new knowledge should aid in creating new and effective acaricidal chemicals. Furthermore, this plant extract’s acaricide activity and its effect on oviposition and larval mortality were established in this work for the first time, indicating the possible use of this extract in the management of ticks.

## 1. Introduction

Ticks negatively impact livestock because they are hematophagous ectoparasites [1]. *Rhipicephalus microplus* is one of the most extensively dispersed ticks in tropical and subtropical areas that is a concern for the livestock industry. The economic impact of this tick species on cattle production worldwide is estimated to be USD 30 billion annually [2,3]. Tick infestations can cause anemia, damage the hide, and spread diseases, including *Anaplasma marginale*, *Babesia bovis*, and *Babesia bigemina* [4].

Currently, various synthetic acaricides are used to control *R. microplus*. However, the continuous use of these acaricides has resulted in the selection of acaricide-resistant and multi-drug-resistant tick populations [5,6,7]. Plant extracts and oils may be a viable alternative to conventional pesticide approaches that have been overused and misused [8]. Plant extracts are associated with lower environmental and food contamination, result in slower development of arthropod resistance, and are of lower toxicity to animals and humans [9].

As a source of unrivaled chemo-diversity, medicinal plants’ pure chemicals and standardized extracts provide a bountiful supply of potential novel drugs. Modernization, standardization, quality control, and greater understanding of ethnomedicinal plant medicines’ active components and modes of action will affect their international acceptance. Steroids, alkaloids, terpenes, flavonoids, phenylpropanoids, amides, and lignans are among the plant metabolites that have drawn the most attention as potential bioactive compounds against ticks [10]. In addition, products from several angiosperm plant species have been used to control economically important tick species [11,12,13,14,15], including acaricide-resistant species such as organophosphate and pyrethroids-resistant *R. microplus* [16,17].

*Datura innoxia* Mill. (Solanaceae), commonly known as devil’s trumpet or thorn-apple, is a plant with ethnopharmacological significance and is native to China, Mexico, the United States, the Caribbean Islands, and Asia [18]. The plant holds a unique position in Ayurveda and has been used to treat various ailments, including rabies, leprosy, and other infectious diseases [19]; however, acute toxicity and delirium may occur if the extract is used without caution. In addition to atropine and other tropanes, *D. innoxia* contains scopolamine hyoscyamine and withanolides (lactones). When seed extracts from several Datura species were tested for their ability to neutralize the stable diphenylpicrylhydrazyl (DPPH) radical and scavenge free radicals, *D. innoxia* showed the highest antioxidant capability [20]. Recently, a novel dinoxin B and anolide isolated from methanol extracts of *D. innoxia* leaves displayed IC50 values in the micromolar range against multiple human cancer cell lines [21].

Multiple functions of the Glutathione S-transferases (GSTs; EC 2.5.1.18) tick enzyme include the degradation of toxic chemicals, the oxidative stress response, and the binding of intracellular ligands such as heme and porphyrin [22]. When consuming blood, parasites have developed several ways to minimize oxidative damage and control redox homeostasis; to avoid heme poisoning, ticks have highly active antioxidant systems in which antioxidant enzymes like Glutathione S-transferases (GST) play an essential role [22,23,24].

Redox balance mediated by GST also plays a significant role in the tick’s survival ability in the face of acaricides [25,26,27,28]. *D. innoxia* aerial component extracts may have anti-tick properties yet to be discovered. *D. innoxia* plant extract was used in this study to test its in vitro acaricidal efficacy on *R. microplus* larvae and engorged females. Further, in silico docking was used to investigate the molecular inhibition profiles of the more potent phytochemicals against *R. microplus* GST activity, all to limit tick acaricide resistance.

## 2. Materials and Methods

### 2.1. Preparation of Plant Extracts

Aerial plant leaves of *D. innoxia* were obtained from district Mardan of Khyber Pakhtunkhwa (KPK) province, Pakistan. The plant leaves were rinsed in running tap water to clean them from debris. The plant was then identified and deposited in the herbarium of the Department of Botany, Abdul Wali Khan University Mardan (AWKUM) for identification. The plant was verified and allotted an accession number Awkum. Bot. 223.9.7. The crude ethanolic extract of *D. innoxia* leaves was made by the maceration method according to the published procedure [29]. *D. innoxia* leaves were air dried for 2 weeks at room temperature (30 °C) and then pulverized into powder using a plant grinder (Panasonic Model MX-AC210, Osaka, Japan). *D. innoxia* leaf powder was weighed, and 50 g was added to 600 mL (1:12) of 96% ethanol and stored at room temperature (25 ± 3 °C). The solution was then agitated for 48 h in an orbital shaking incubator (Cole-Parmer Model EW-51700-14, Cole-Parmer, Vernon Hills, IL, USA) at 200 RPM. The extract was then concentrated for 24 h in a water bath (Model WTB15, Memmert GmbH and Co. KG, Schwabach, Germany) to obtain less than 10% of the original solution. The stock solution was refrigerated at −4 °C for further analysis. The stock solution was then diluted in distilled water to create different concentrations of 40, 20, 10, 5, and 2.5 mg/mL (*w*/*v*). Distilled water was used as a negative control, and trichlorfon as a positive control.

### 2.2. Collection of R. microplus Ticks

Totally engorged females of *R. microplus* from natural infestation were collected from the ground and cattle bodies kept on farms in and around the Mardan area. After collection, the ticks were cleaned using water to remove dirt and dried with absorbent paper. For oviposition, 200 ticks were transferred to Petri dishes (with holes in the lid allowing air to circulate) and incubated in laboratory conditions (28 ± 1 °C and 85 ± 5% relative humidity). The deposited eggs were transferred to fresh glass vials after complete egg laying and held for hatching under similar incubation circumstances for 20 days. The larval packet test (LPT) was performed on larvae aged 14 to 15 days. The remaining ticks, were used in the adult immersion test (AIT).

### 2.3. Adult Immersion Test (AIT)

The AIT was performed with minimal adjustments as described by [17]. Several concentrations of the extract were used, including 2.5, 5, 10, 20, and 40 mg/mL. A total of 75 (5 × 7 × 3 = 105) engorged adult ticks were used in this study. Three replicates were employed, each separately with five ticks at each concentration, keeping in mind that all the ticks in a replicate were collected from the same farm [30]. Engorged females in each replicate group were weighted. Adult female ticks were submerged in 1 mL of the extract concentrations for 5 min. The treated 5 ticks were then placed in a Petri dish with Whatman filter paper no. 1, and the dish was maintained in an incubator (Model BIBD-101, Toronto, ON, Canada), which was adjusted to 28 °C and 85 ± 5% RH. The weight of eggs laid in each replicate group was obtained. For surviving ticks, data on the influence of plant extracts on oviposition (egg-laying) inhibition was collected and analyzed as described by [31]. To determine the extract’s effectiveness against engorged females, the engorged female’s weight was measured, and the predicted mass of eggs produced by the female was then used to calculate the percent egg inhibition (inhibition of oviposition (% IO)) by using the following formula [32], with and without treatment:

$$\%\;\mathrm{IO}=\frac{\mathrm{IE}\;\mathrm{control}\;\mathrm{group}-\mathrm{IE}\;\mathrm{treated}\;\mathrm{group}}{\mathrm{IE}\;\mathrm{control}\;\mathrm{group}}\times 100$$

where the index of egg laying IE = mean weight of eggs laid ÷ mean weight of engorged females.

### 2.4. Larval Packet Test (LPT)

A bioassay was performed using 2.5, 5, 10, 20, and 40 mg/mL of ethanol extracts. A packet of 100 larvae were immersed in 200 μL of each extract concentration in 1.5 mL centrifuge tubes. Ten minutes after, the solution was pipetted out, and the entire tube was dried by absorbing the fluid with a filter paper strip. The tubes were then covered with cotton cloths and secured with rubber bands. Three replicates with each extract concentration were performed. The larval packets treated with LPT were put into the incubator at the same temperature and relative humidity described above. Larval mortality was determined by counting the number of live (only larvae that could walk were considered alive) and dead larvae (larvae without movement, ataxia, or movement only of appendages were considered dead) after 24 and 48 h.

### 2.5. Glutathione S-Transferases Protein Sequence of R. microplus

The protein sequence of glutathione S-transferases (RmGST) was obtained from the UniProt KB database, a publicly available library of protein sequences with comprehensive functional annotations. This particular protein’s accession number is E5L876.

### 2.6. Modeling the 3D Structure of RmGST

TrRosetta (“https://robetta.bakerlab.org/ (accessed on 1 August 2022)”), a protein prediction server based on continuous automated model assessment (CAMEO, “https://www.cameo3d.org/ (accessed on 1 August 2022)”), performed the 3D structure predictions (de novo or ab initio). TrRosetta predicts protein structure using a modeling technique based on deep machine learning [33].

### 2.7. Modeled Structure Validation

The verification of the stereochemical quality of the modeled structure of the query protein was made using the PROCHECK (“https://www.ebi.ac.uk/thornton-srv/software/PROCHECK (accessed on 5 August 2022)”), Verify3D (“http://servicesn.mbi.ucla.edu/Verify3D/ (accessed on 5 August 2022)”), and ERRAT (“https://servicesn.mbi.ucla.edu/ERRAT/ (accessed on 5 August 2022)”) servers.

### 2.8. Determining Binding Sites

To assess the potential binding sites of the selected proteins, the Computed Atlas of Surface Topology of Proteins (CASTp) was employed [34]. PyMOL (PyMOL Molecular Graphics System, Version 1.5.0.4, Schrödinger, LLC, New York, NY, USA) and Chimera 1.16 were used to visualize the predicted sites. The predicted binding sites with constrained areas and volumes, where no ligands could fit, were ignored [35].

### 2.9. Ligands, Protein Preparation for Molecular Docking

A PubChem database (“https://pubchem.ncbi.nlm.nih.gov/ (accessed on 8 August 2022)”) search was undertaken to identify compounds from *D. innoxia*; 21 compounds were found and selected. ChemDraw 16.0 was used to create and analyze the 2D structures (.mol) of all 21 compounds. Chem3D 16.0 transforms all of the chemicals into 3D designs (.pdb). All rotatable bonds in ligands were made flexible, and Gasteiger atomic partial charges were assigned using the AutoDockTools program [36]. In preparation for receptor docking using the AutoDockTools program, all water molecules were removed, the co-crystallized ligand was removed, Gasteiger atomic partial charges were assigned, and all receptors and ligands were converted to the PDBQT format. The grid box was constructed using 56, 56, and 40 grid points in the x, y, and z axes, respectively, with a grid point spacing of 0.347 Å. The coordinates of the main grid box are 33.494, 21.231, and −12.276. Nine alternative conformations were created for each ligand and ranked based on their binding energies using AutoDockVina scoring functions (https://vina.scripps.edu (accessed on 8 August 2022)). The Discovery studio visualizer (version 19.1.0.18287 BIOVIA, San Diego, CA, USA) was used for the post-docking experiments. The Discovery studio visualizer selected the conformations with the lowest (most advantageous) free binding energy to examine the interactions between the target receptor and ligands.

### 2.10. Statistical Analysis

All statistical analyses were made using R Statistical software (version 4.1.3) [37] and RStudio software (version 2022.12.0+353) [38]. One-way ANOVA followed by post hoc Tukey’s honesty significance difference (HSD) test was performed using the “agricolae” package to find the significant difference between larval mortalities at 24 and 48 h time intervals and % IO for different concentrations [39]. The lethal concentrations causing 50% and 90% mortalities (LC_50_ and LC_90_) (24 and 48 h) [40] and lethal time causing 50% mortality (LT_50_) for the ethanolic extract were calculated using probit analysis [41] by applying the R “ecotox” package [42], where the heterogeneity significance (*p*-value) was kept at 0.05, and the fiduciary confidence limit was set at 95%. All results were visually presented by the “ggplot2” package [43].

## 3. Results

### 3.1. Adult Immersion Test (AIT)

**Figure 1:**
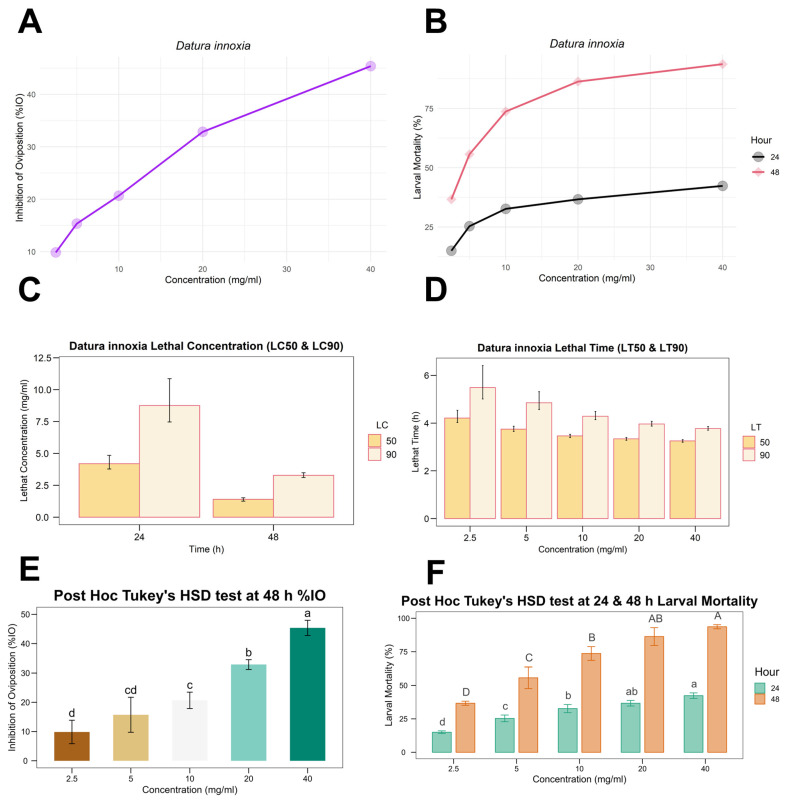
(**A**) Response curve graph for the inhibition of oviposition (% IO in AIT) at 48 h; (**B**) concentration–mortality curve for larval mortality at 24 and 48 h in LPT; (**C**,**D**) represent log-transformed LC_50_, LC_90_, LT_50_, and LT_90_ with error bars showing the corresponding log-transformed lower and upper confidence limits values of the LC and LT in LPT; (**E**,**F**) represent one-way ANOVA with post hoc Tukey HSD test for % IO and larval mortality, for AIT and LPT, respectively.

Table 1 displays the percentages observed for the in vitro effectiveness of *D. innoxia* ethanolic extract against *R. microplus*. According to Table 1 and Figure 1A,E, the % IO increased as extract concentration increased (from 9.86% IO at 2.5 mg/m to 45.37% IO at 40 mg/mL) at 48 h. Overall, with higher concentrations (≥5 mg/mL) and a longer treatment time (48 h), the plant extract could significantly increase larval mortality and have the potential to hamper the egg-laying activity of *R. microplus*.

### 3.2. Larval Packet Test (LPT)

*D. innoxia* plant extract showed significant larvicidal activity, with more than 50% larval mortality at all concentrations except 2.5 mg/mL after 48 h, comparable to the mortality induced by the positive control, trichlorfon. The LC_50_ value of the extract at 48 h of treatment is 4.06 (3.53–4.58) mg/mL, and its corresponding LC_90_ is 26.63 (22.56–32.56) mg/mL (Table 2), Figure 1C, whereas its LT_50_ value is 25.94 (24.53–27.26) h and its LT_90_ values are 43.47 (40.57–47.46) h at 40 mg/mL concentration (Table 3 and Figure 1D). The significant difference between different concentrations for mortality at 24 h and 48 h and % IO at 48 h treatment by post hoc Tukey’s HSD test is shown in Figure 1F.

### 3.3. Analysis of Protein Models and Validation

**Figure 2:**
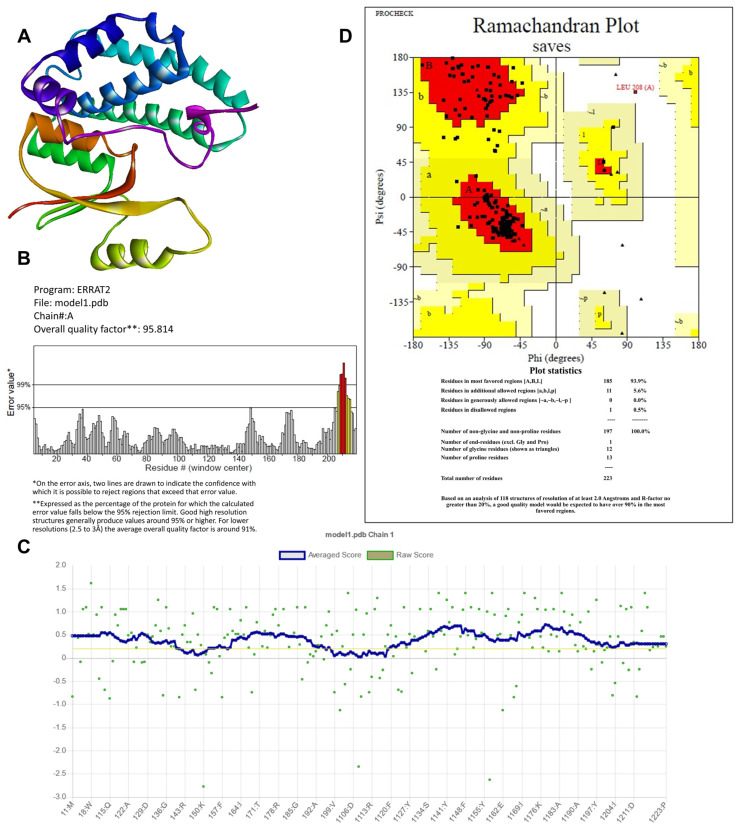
(**A**) *Rhipicephalus microplus* glutathione S-transferases (RmGST) three-dimensional (3D) structure predicted by the trRosetta server; (**B**–**D**) ERRAT, Z-scores, and Ramachandran map, respectively, validate the protein structure and amino acid position of RmGST. The Ramachandran plot of RmGST indicates the percentage of residues in favored regions (red) and allowed regions (yellow) where the bars in the ERRAT plot represents the error value (white: error < 95%, yellow: error < 99%, and red: error > 99%).

The amino acid sequence of the target protein, Glutathione S-transferases (RmGST) UniProt identifier E5L876, comprising 223 amino acid residues, was used to predict a full-length model structure using the trRosetta server template-free modeling (de novo or ab initio) technique (Figure 2A). This server has available five model structures. The models from trRosetta appear superior due to their domain area architecture. The predicted 3D structures were validated and quality-evaluated to produce suitable protein structures. The correctness of the models is determined by the Ramachandran plots produced by ERRAT, Verify3D, and PROCHECK.

The validation scores of model 1 from trRosetta are the preferred model among the RmGST protein structures. ERRAT generated the model’s quality factor of 95.814% (Figure 2B). Two lines were put on the error axis to represent the degree of certainty with which the regions that exceeded the error value may be rejected. It is presented as a percentage of the protein for which the estimated error value is less than the rejection level of 95%. Structures generating quality factors of 95% and higher are considered high resolution. The average quality factor for lower resolution (2.5–3.0 Å) is roughly 91%. By comparing highly enhanced predicted structures to statistical analysis, ERRAT provided the value of the error function and revealed confidence limits. As a result, 95.814% of the total value is considered significant and accurate.

The PROCHECK service evaluated the stereochemical quality of protein structures on a residue-by-residue basis and overall structural geometry. Ramachandran plots revealed the U and W distributions of non-glycine and non-proline residues and the distribution of residues (Figure 2D). To distinguish between favorable and unfavorable regions, the phi and psi angles were plotted against each other. These perspectives were used to assess the quality of different regions. PROCHECK results revealed that 93.9% of amino acid residues were in preferred areas, 5.6% were in allowed regions, 0.0% were in the generous region, and 0.5% were in prohibited areas (Figure 2D). The Verify-3D application confirmed the model’s stereochemical quality [44]. Residues with a score function more significant than 0.2 are regarded as acceptable. Furthermore, 85.2% of the residues received a score greater than 0.2. The results verify the high quality of the projected model [45], as shown in Figure 2C.

### 3.4. Active Site Prediction

**Figure 3:**
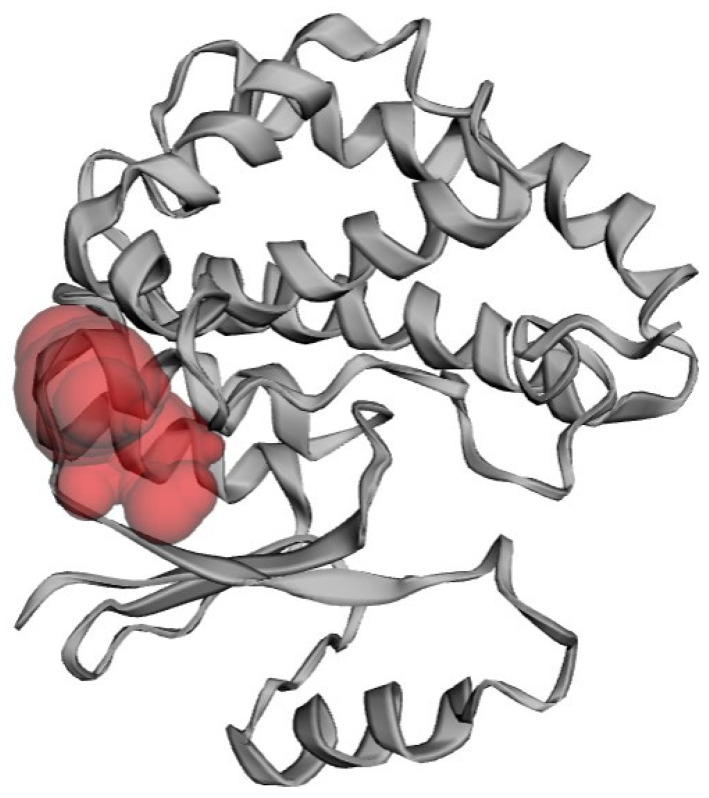
Determination of RmGST active sites using the CASTp server.

CASTp was utilized to identify potential binding sites within the RmGST protein structure (Figure 3). Ligand binding sites on proteins tend to include the most prominent pockets or cavities [45,46,47]. Hence, pockets with relatively low areas and volumes, where no ligand could fit, were not examined [48,49]. The target protein’s active site consisted of the amino acids ARG18, LEU21, ALA22, ASP25, ALA26, LYS27, VAL28, ASP30, HIS193, ALA196, TYR197, SER200, and LYS202.

### 3.5. Docking Studies

After validating the RmGST three-dimensional (3D) structural model and defining the target pocket, the parameters for the docking analysis were determined using AutoDockVina software (version 4.2.6). The docking stage was used to find the ligands having the best interactions with protein residues. AutoDockVina was used for docking the selected chemicals and ranking complexes based on their greatest binding affinities. ChemDraw created and reduced the 2D structures of the 21 specified chemicals. The complex was chosen for its high binding affinity and examined for interactions between RmGST residues.

**Figure 4:**
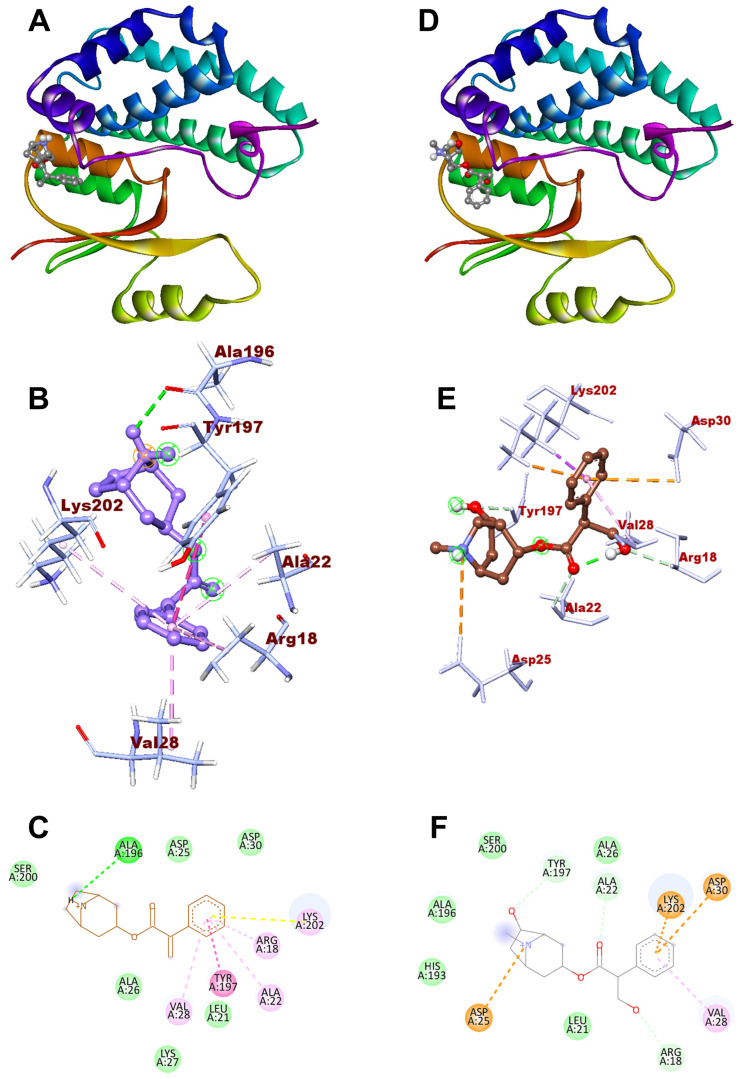
(**A**,**B**) Docking of norapoatropine with binding sites of RmGST’s 3D complex structure and (**C**) their 2D interaction. (**D**,**E**) Docking of 7-hydroxyhyoscyamine with binding sites of RmGST’s 3D complex structure and (**F**) their 2D interaction.

*D. innoxia*’s phytochemicals revealed a higher docking score at the active sites of the RmGST protein structure in molecular docking studies. All of the compounds demonstrated binding affinity within the co-crystallized ligand binding sites. The compound Norapoatropine shows the highest binding affinity of −7.3 Kcal/mol, producing one hydrogen bond connection with Ala196 and one residual hydrophobic interaction with Arg18, Ala22, Val28, Tyr197, and Lys202. Similarly, the compound 7-Hydroxyhyoscyamine also showed the maximum binding affinity of −7.0 Kcal/mol and showed persistent hydrophobic interactions with the amino acids Arg18, Ala22, Asp25, Val28, Asp30, Tyr197, and Lys202. In silico docking studies demonstrated that Norapoatropine and 7-Hydroxyhyoscyamine had high docked scores (−7.3 and −7.0 Kcal/mol, respectively), indicating that these compounds may be potent inhibitors of *R. microplus* RmGST protein (Figure 4).

### 3.6. Molecular Dynamics Simulation

**Figure 5:**
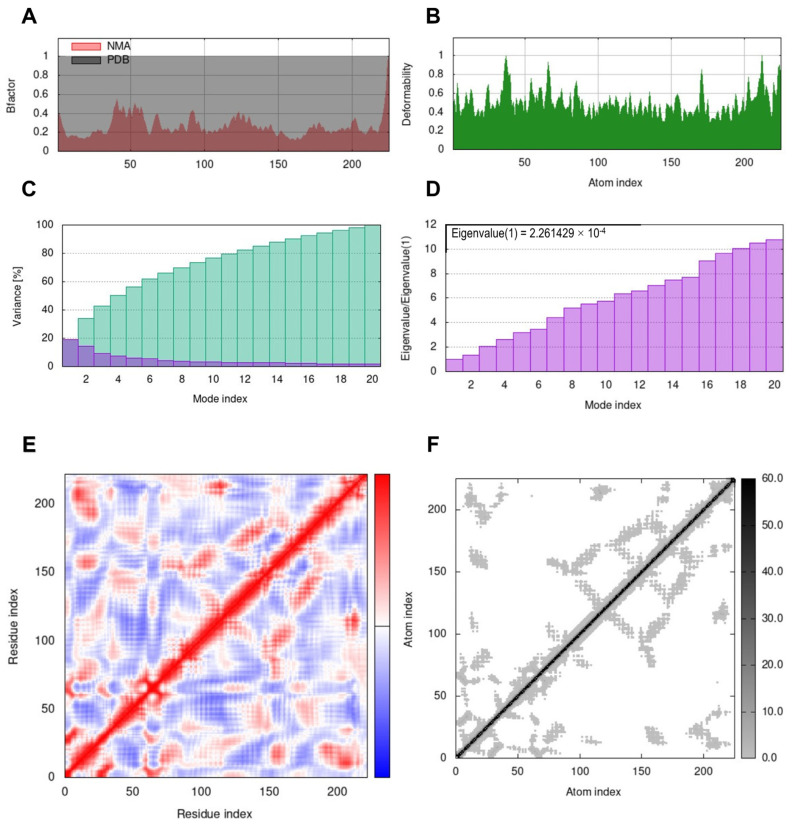
Result outputs of the normal mode analysis (NMA) through the iMODs server. (**A**) factor; (**B**) deformation plot; (**C**) variance plot; (**D**) eigenvalue; (**E**) covariance matrix plot; (**F**) elastic network model.

The iMODS server was used to perform normal mode analysis (NMA) on proteins to investigate their stability. The complex’s deformability is determined by the individual deformation of each residue, which is represented by hinges in the chain (Figure 5B). The calculated eigenvalue, which indicates the complex’s motion stiffness, was 2.2614 × 10^−4^ (Figure 5D). An inverse connection was discovered between the eigenvalue and the variance of each normal mode (Figure 5C). The NMA-derived B-factor scores were comparable to the RMS (Figure 5A). The covariance matrix depicted the coupling between pairs of residues, with various pairings exhibiting correlated, anti-correlated, or uncorrelated movements, denoted by red, blue, and white, respectively (Figure 5E). Finally, an elastic network model (Figure 5F) was built, and it displayed the pairings of atoms linked by springs based on the degree of stiffness between them, with stiffer strings appearing as darker gray.

## 4. Discussion

Plants have an essential role in traditional medicine, and they contain several compounds that may be able to interfere with the metabolic processes and life cycle of ticks [50]. Rosado-Aguilar et al. evaluated the possible use of plant extracts to manage arthropods of veterinary importance; a few plants were found to be promising against ticks [51]. The acaricidal activity of the *D. innoxia* plant extract was discovered in this study, and this finding can contribute to the development of plant-based acaricides for tick management.

The in vitro components of this study show that the *D. innoxia* extract exhibits acaricidal properties directed at two phases of *R. microplus* development: larvae and adult females with higher concentrations (≥5 mg/mL) and a longer treatment time (48 h). These findings are consistent with previous research. A 95% ethanol extract from *D. stramonium* leaves generated 20% mortality against *R. microplus* within 72 h of treatment in an adult immersion bioassay [52]. Additionally, in vitro investigations revealed that the 95% methanol extract of *D. stramonium* suppressed *R. microplus* oviposition by 77% [30]. Leaf and seed extracts of *D. stramonium* produced 98% and 25% mortalities of spider mites (*Tetranychus urticae Koche*) after 24 h, respectively, where the death rate of *T. urticae* females rose when the concentration of leaf extract was increased from 2416 to 7250 ppm, but there was no change when the amount of seed extract of the same concentration was increased [53]. In this study, *D. innoxia* extracts killed the *R. microplus* larvae (73.67–93.67% at 10–40 mg/mL), and the concentration 40 mg/mL was similar to the positive control group (*p* < 0.0). *D. innoxia* extracts showed a strong effect, producing the inhibition of oviposition of engorged females (IO 15.73–45.37% at 5–40 mg/mL) compared with the negative control (IO 0.20%). 

Computer modeling approaches are widely used in drug design. Computer-aided drug design is a common term for this approach, whereas “structure-based drug design” refers to drug development based on the 3D structure of the biomolecular target. This kind of drug design relies on an increasing number of computational programs developed to improve the affinity and stability of these protein-based therapeutics [54,55,56]. To predict the binding mechanisms of these drug-like compounds, 21 chosen compounds from the *D. innoxia* plant were docked to the target protein *R. microplus* Glutathione S-transferases using AutoDock Vina software. The findings revealed that the chemicals norapoatropine and 7-hydroxyhyoscyamine had significant interactions with active site residues of the target protein RmGST, making them a suitable RmGST inhibitor and comparable to reference ligands. Norapoatropine, with a docking score of −7.3 Kcal/mol among the 21 different docking compounds, was determined to be the most effective of the investigated compounds, followed by 7-Hydroxyhyoscyamine with a docking score of −7.0 Kcal/mol. Both of these compounds have good inhibitory activity and their docking score is in the region of certain standard ligands, such as Trichlorofon (−4.1 Kcal/mol). Furthermore, both these ligands exhibited good interaction with *R. microplus RmGST* protein. The most potent ligand, norapoatropine, formed one hydrogen interactions with a bond length of 1.461 Å with the Ala 196 active amino acid residue and hydrophobic (Pi-alkyl) interaction with Arg 18, Ala 22, Val 28, and Lys 201, and one Pi-Pi T shaped interaction with Tyr 127 amino acid residue. 7-Hydroxyhyoscyamine also demonstrated a higher binding affinity with residual amino acids compared with Trichlorofon, which formed three-carbon–hydrogen bond Arg 18, Ala 22, and Tyr 127 and three-Pi–cation bond with Asp 25, Asp 30 and Lys 202, and one Pi-alkyl bond Val 28 amino acid residue. The results of in silico docking showed that the compounds norapoatropine and 7-Hydroxyhyoscyamine had the highest docked scores (−7.3 and −7.0 Kcal/mol, respectively), compared to other compounds, and similar residual interactions to those of Trichlorofon within the binding pocket, indicating that these compounds are promising anti-tick agents against *R. microplus*. In summation, the current work establishes a computational basis for RmGST inhibitors. Future studies should focus more closely on the therapeutic efficacy of these chemicals, their method of action on ticks, and their proteins, thereby enabling the development of new tick control resources.

## 5. Conclusions

This study proved that plant extract from *D. innoxia* has in vitro acaricidal action against larvae and engorged females of *R. microplus*. Our results will help verify the conditions that stimulate acaricidal activity. In addition, the examined extracts may be helpful in the creation of a long-term strategy for tick management in the livestock industry due to their acaricidal characteristics. The in vitro bioassays and in silico methodologies explored in this study provide an opportunity to evaluate acaricidal effects by merging different disciplines. This study opens the opportunity for tick management in the cattle industry. Additional research must be done to assess their impact on the adult tick stage and acaricidal activity in vivo.

## Figures and Tables

**Table 1:** Mean % larval mortality and inhibition of oviposition at different concentrations of *D. innoxia* plant extract against *R. microplus* in vitro in LPT and AIT, respectively.

Extract	Concentration (mg/mL)	% Mean ± Standard Deviation		
		% Larval Mortality		% Inhibition of Oviposition (IO)
		24 h	48 h	
*D. innoxia*	40	42.33 ± 2.08 ^b^	93.67 ± 1.53 ^a^	45.37 ± 2.60 ^b^
	20	36.67 ± 2.08 ^bc^	86.33 ± 6.66 ^ab^	32.85 ± 1.63 ^c^
	10	32.67 ± 3.06 ^c^	73.67 ± 5.13 ^b^	20.68 ± 2.80 ^d^
	5	25.33 ± 2.52 ^d^	55.67 ± 8.02 ^c^	15.73 ± 5.98 ^de^
	2.5	15.00 ± 1.00 ^e^	36.67 ± 1.53 ^d^	9.86 ± 4.01 ^ef^
Control Group	Trichlorfon	58.00 ± 3.00 ^a^	97.67 ± 2.52 ^a^	83.74 ± 5.02 ^a^
	Distilled water	0 ^f^	1.00 ± 1.00 ^e^	0.20 ± 1.46 ^f^

Means not sharing any letters in the same column were significantly different by Tukey’s HSD test at a 5% significance level (*p* < 0.05).

**Table 2:** Medium lethal concentration causing 50% and 90% mortalities (LC_50_ and LC_90_ values) of *D. innoxia* plant leaf extract against *R. microplus* in vitro.

Time (h)	LC_50_ (mg/mL)	95% Confidence Limits		LC_90_ (mg/mL)	95% Confidence Limits		Slope ± S.E.	Intercept ± S.E.	Chi-Square (χ^2^)	*p*-Value
		LCL	UCL		LCL	UCL				
24	66.44	43.79	127.55	6310.72	1754.24	52,277.92	0.64 ± 0.08	−1.18 ± 0.09	6.48	0.93
48	4.060	3.530	4.586	26.632	22.564	32.56	1.56 ± 0.09	−0.95 ± 0.09	16.51	0.22

LCL: low confident limit, UCL: upper confident limit, S.E.: standard error.

**Table 3:** Lethal time causing 50% and 90% mortalities (LT_50_ and LT_90_ values) at varying concentrations for *D. innoxia* against *R. microplus* in vitro.

Concentration (mg/mL)	LT_50_ (h)	95% Confidence Limits		LT_90_ (h)	95% Confidence Limits		Slope ± S. E	Intercept ± SE	Chi-Square (χ^2^)	*p*-Value
		LCL	UCL		LCL	UCL				
2.5	67.39	56.21	93.62	241.63	150.14	608.63	2.31 ± 0.38	−4.22 ± 0.59	0.35	0.98
5	42.46	38.67	47.99	127.75	96.59	203.91	2.67 ± 0.35	−4.36 ± 0.55	5.88	0.21
10	31.99	29.78	34.25	72.7173	63.12	89.09	3.59 ± 0.35	−5.41 ± 0.55	3.56	0.47
20	28.28	26.62	29.88	52.51	48.17	58.76	4.77 ± 0.38	−6.92 ± 0.58	7.88	0.09
40	25.94	24.53	27.26	43.47	40.57	47.46	5.71 ± 0.47	−8.08 ± 0.65	1.14	0.89

LCL: low confident limit, UCL: upper confident limit, S.E.: standard error.

## Data Availability

Not applicable.
